# Automatic segmentation and classification of frontal sinuses for sex determination from CBCT scans using a two-stage anatomy-guided attention network

**DOI:** 10.1038/s41598-024-62211-y

**Published:** 2024-05-23

**Authors:** Renan Lucio Berbel da Silva, Su Yang, DaEl Kim, Jun Ho Kim, Sang-Heon Lim, Jiyong Han, Jun-Min Kim, Jo-Eun Kim, Kyung-Hoe Huh, Sam-Sun Lee, Min-Suk Heo, Won-Jin Yi

**Affiliations:** 1 Discipline of Oral Radiology, Department of Stomatology, School of Dentistry, University of São Paulo, São Paulo, SP Brazil; 2 Department of Applied Bioengineering, Graduate School of Convergence Science and Technology, Seoul National University, Seoul, 08826 South Korea; 3 Interdisciplinary Program in Bioengineering, Graduate School of Engineering, Seoul National University, Seoul, 08826 South Korea; 4 Department of Electronics and Information Engineering, Hansung University, Seoul, 02876 South Korea; 5 Department of Oral and Maxillofacial Radiology and Dental Research Institute, School of Dentistry, Seoul National University, Seoul, 03080 South Korea

**Keywords:** Deep learning, Sex determination, Frontal sinus, CBCT, Anatomy-guided attention, Machine learning, Image processing, Dental radiology, Forensic dentistry

## Abstract

Sex determination is essential for identifying unidentified individuals, particularly in forensic contexts. Traditional methods for sex determination involve manual measurements of skeletal features on CBCT scans. However, these manual measurements are labor-intensive, time-consuming, and error-prone. The purpose of this study was to automatically and accurately determine sex on a CBCT scan using a two-stage anatomy-guided attention network (SDetNet). SDetNet consisted of a 2D frontal sinus segmentation network (FSNet) and a 3D anatomy-guided attention network (SDNet). FSNet segmented frontal sinus regions in the CBCT images and extracted regions of interest (ROIs) near them. Then, the ROIs were fed into SDNet to predict sex accurately. To improve sex determination performance, we proposed multi-channel inputs (MSIs) and an anatomy-guided attention module (AGAM), which encouraged SDetNet to learn differences in the anatomical context of the frontal sinus between males and females. SDetNet showed superior sex determination performance in the area under the receiver operating characteristic curve, accuracy, Brier score, and specificity compared with the other 3D CNNs. Moreover, the results of ablation studies showed a notable improvement in sex determination with the embedding of both MSI and AGAM. Consequently, SDetNet demonstrated automatic and accurate sex determination by learning the anatomical context information of the frontal sinus on CBCT scans.

## Introduction

Sex determination is fundamental in establishing the biological profile of unidentified humans^1^. Accurate individual identification is important to society, particularly sexual dimorphism, as it has significant implications in criminal investigations^1,2^. Skeletal remains of the human body can preserve their shapes and structures even in harsh environments and disaster events, making them suitable for sex determination^3^. Manual morphological measurement and analysis of skeletal remains such as the hand bone^4^, hip bone^5^, teeth^6^, mandible^7^, skull^7^, and paranasal sinus^8,9^ are widely used to determine the sex of individuals. These methods are based on the presence of significant differences in the sizes and shapes of the teeth, pelvis, and skull between males and females^10^. Skull structures, including the paranasal sinuses, teeth, and cranial suture patterns can potentially be used to identify an individual.

The frontal sinuses, which are part of the paranasal sinuses of the head, are cavities located inside the frontal bone and can be used as an indicator of sex due to their unique sizes, shapes, and patterns in males and females^10–13^. The frontal sinus completes growth by around the 20th year and remains relatively unchanged throughout adulthood, making it ideal for postmortem identification as well. The unique and stable morphology of the frontal sinuses makes them an important and reliable tool for forensic identification purposes. Frontal sinus imaging can be done using a variety of techniques such as X-ray, computed tomography (CT), cone-beam computed tomography (CBCT), and magnetic resonance imaging (MRI)^10–15^. Several studies have reported success using frontal sinus imaging for individual identification, notwithstanding the challenge of establishing a universally accepted and objective standardized method. Identification of sexual dimorphism in the frontal sinuses has made sex determination based on this anatomical structure a valuable tool to reduce the range of possibilities to be considered during individual identification, hence aiding in the creation of a more dependable biological profile of human remains^10^. CBCT is widely used in the field of dentistry as it provides accurate and detailed three-dimensional imaging of the maxillofacial region. Its advantages over other imaging techniques have made it a valuable tool in various dental specialties, including orthodontics, implant dentistry, endodontics, and even forensic dentistry^16–18^. Several studies have demonstrated the effectiveness of manual analysis of the paranasal sinuses (such as frontal sinus and maxillary sinus) from CBCT images for sex determination, with a reported accuracy of 80.0% for manual identification^19,20^.

In recent years, deep learning has been applied to medical image analysis tasks including image classification, detection, segmentation, denoising, and synthesis^21,22^. Several studies have reported methods based on deep learning for sex determination from CT or CBCT images. Bewes et al. proposed a sex determination method based on an artificial neural network on CT images^23^. This artificial neural network was trained on a dataset containing 900 skulls reconstructed from CT images and showed 95% accuracy for sex determination. Baban et al. reported a machine learning-based method that used morphometric measurements of the mandible on CBCT images as input^24^. This method showed 90% accuracy using Gaussian Naive Bayes. Senol et al. also proposed a machine learning-based method for sex determination using dental parameters of the maxillary molar and canine teeth obtained from CBCT images^25^. The method achieved 81% accuracy in sex determination with the ADA Boost Classifier algorithm. Although several studies have reported machine learning-based methods for sex determination, these approaches require several steps including manual segmentation of the skull, manual measurement of dental parameters, feature extraction, and classification; each of these steps is labor-intensive, time-consuming, and error-prone^26^. Moreover, existing methods face find it challenging to discriminate subtle differences in shapes and sizes of the frontal sinus on CBCT scans between males and females. Therefore, an automatic and accurate method for sex determination from CBCT scans is required. To the best of our knowledge, no previous study has performed fully automated segmentation and classification of the frontal sinuses for sex determination on CBCT scans using deep learning.

The purpose of this study was to automatically and accurately determine sex from a CBCT scan using a two-stage anatomy-guided attention network (SDetNet). Our main contributions are as follows: (1) The proposed SDetNet was designed to automatically and accurately segment the frontal sinus and predict the sex from a CBCT scan. The first stage deep learning model was a 2D frontal sinus segmentation network (FSNet) that segmented the frontal sinus on CBCT images and extracted regions of interest (ROIs) near it. The second stage was a 3D anatomy-guided attention network (SDNet) for accurate sex determination from a CBCT scan. (2) We introduced multi-channel inputs (MCI) and an anatomy-guided attention module (AGAM) to improve the performance of sex determination. The proposed MCI and AGAM encouraged SDetNet to learn the anatomical context of the frontal sinus for accurate and robust prediction of sex from a CBCT scan. In addition, we demonstrated the effectiveness of the AGAM and MCI by an experimental ablation study.

## Materials and methods

### Data acquisition and preparation

We collected a total of 310 CBCT scans acquired from 310 patients (mean age: 26.81 ± 11.36, 155 males and 155 females) who underwent Seoul National University Dental Hospital from 2020 to 2022. This study was performed with approval from the institutional review board of Seoul National University Dental Hospital (ERI123041). The ethics committee waived informed consent because this was a retrospective study. The study was performed following the Declarations of Helsinki. CBCT scans were acquired using a CS9300 (CS 9300, Carestream Health, Rochester, USA) with voxel sizes of 0.3 × 0.3 × 0.3 mm^3^, dimensions of 640 × 670 × 670 pixels, and 16-bit depth under conditions of 80 or 90 kVp and 8 or 10 mA. All CBCT scans were anonymized and exported in DICOM format. The inclusion criterion was patients aged from 4 to 86 years (Supplementary Fig. S1), while exclusion criteria were patients with visible trauma, previous surgery, or pathological conditions in the frontal region of the skull.

**Figure 1:**
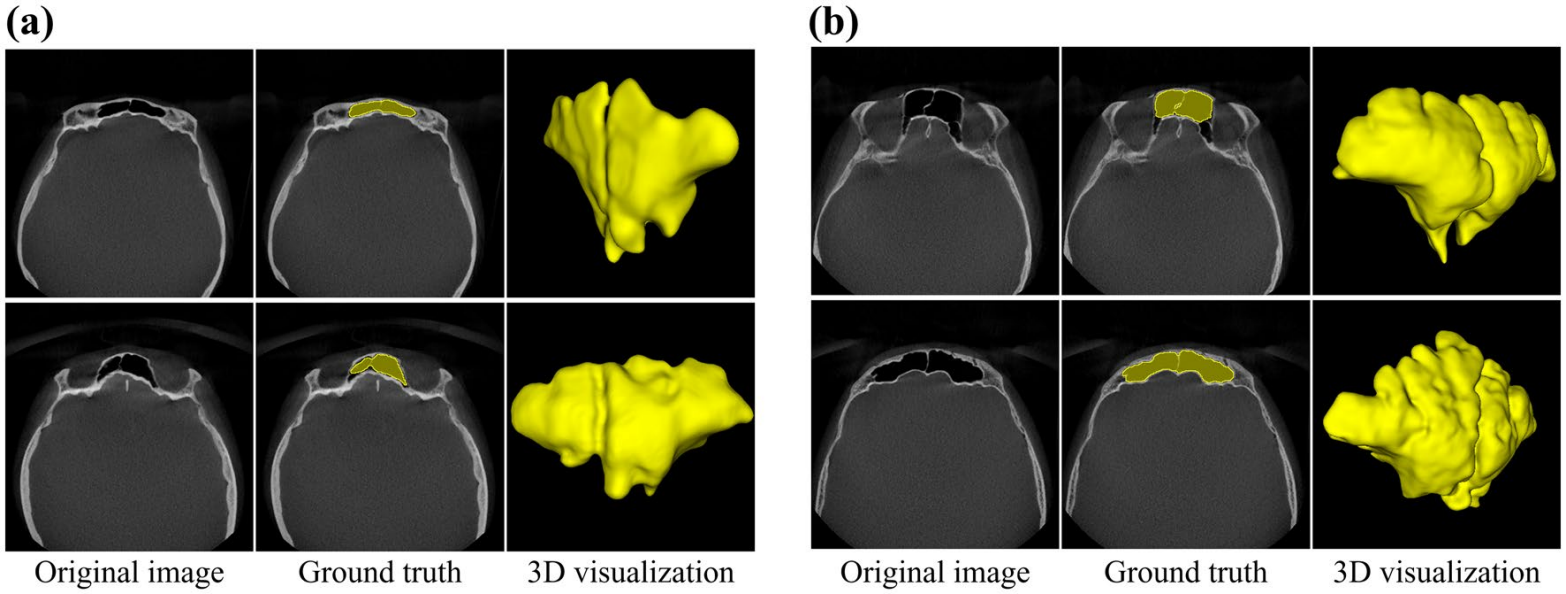
(**a**, **b**) CBCT images with label masks of the frontal sinus acquired from a female and male, respectively.

Among the 310 CBCT scans, we split into 50 and 260 CBCT scans for the frontal sinus segmentation task and the sex determination task, respectively (Table 1). The 50 CBCT scans only used for frontal sinus segmentation were split into 30, 10, and 10 scans for training, validation, and test sets, respectively, and each set had the same sex distribution. Training, validation, and test sets comprised 19,200, 6400, and 6400 CBCT images, respectively. We observed a difference in volume (Supplementary Fig. S2a), length of the major axis (Supplementary Fig. S2b), and length of the minor axis (Supplementary Fig. S2c) between the frontal sinuses of males and females in our dataset. A region of interest (ROI) on a CBCT scan was cropped to 122 × 128 × 128 pixels with the center at the frontal sinus region segmented by FSNet. The 260 CBCT scans only used for sex determination were split into 120, 40, and 100 CBCT scans for training, validation, and test sets, respectively, and each set had the same sex distribution. To generate the ground truth of segmentation masks (Fig. 1a,b), frontal sinus regions on CBCT images were labeled by a radiologist with over five years of experience using 3D Slicer software (www.slicer.org).

**Table 1:** Data configuration for frontal sinus segmentation and sex determination tasks.

Dataset	Number of CBCT images	Number of patients	Number of males	Number of females	Mean age
Frontal sinus segmentation					
Training	19,200	30	15	15	30.2 ± 12.9
Validation	6,400	10	5	5	25.4 ± 2.8
Testing	6,400	10	5	5	32.0 ± 15.2
Sex determination					
Training	76,800	120	60	60	25.5 ± 10.6
Validation	25,600	40	20	20	28.2 ± 11.2
Testing	64,000	100	50	50	27.9 ± 11.5

We estimated the minimum required sample size to detect significant differences in the accuracy of SDetNet and that of other networks when both assessed the same subjects (CBCT scans). We designed the study to capture a mean accuracy difference of 0.05 and a standard deviation of 0.10 between SDetNet and the other networks. Based on an effect size of 0.5, a significance level of 0.05, and a statistical power of 0.80, we calculated a required sample size of N = 128 (G* Power for Windows 10, Version 3.1.9.7; Universität Düsseldorf, Germany). Finally, we split the dataset of CBCT scans into 120, 40, and 100 scans for training, validation, and test sets, respectively.

### The architecture of a two-stage anatomy-guided attention network

**Figure 2:**
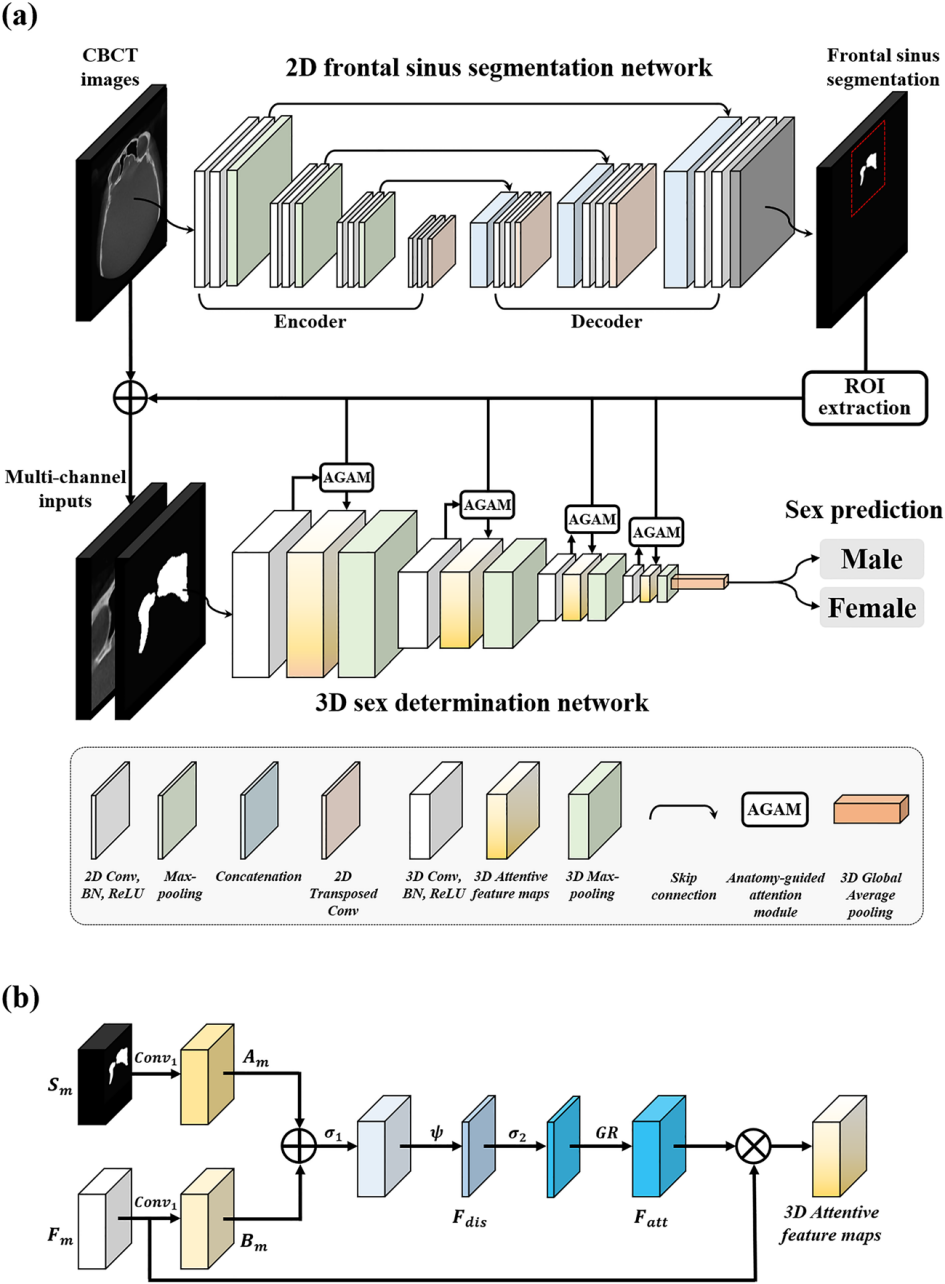
Overview of the proposed two-stage anatomy-guided attention network (SDetNet). (**a**) SDetNet consists of the 2D frontal sinus segmentation network (FSNet) and 3D sex determination network (SDNet). (**b**) The architecture of anatomy-guided attention module (AGAM).

We proposed a two-stage anatomy-guided attention network (SDetNet) for automatic and accurate sex determination from a CBCT scan (Fig. 2). SDetNet consisted of a 2D frontal sinus segmentation network (FSNet) and a 3D anatomy-guided attention network (SDNet). The first stage was 2D frontal sinus segmentation using FSNet, which automatically segmented the frontal sinus regions on CBCT images. Next, 3D sex classification was performed using SDNet, which used the anatomy-guided information from the frontal sinus segmentation to automatically determine the sex of a patient on a CBCT scan. For frontal sinus segmentation on CBCT images, we used FSNet which had a U-shape encoder-decoder architecture with transfer learning. Five popular backbones, namely VGG16^27^, ResNet101^28^, DenseNet201^29^, Inception V3^30^, and EfficientNet-B5^31^ were used as encoders in FSNet. The decoder part had five levels of layers with 2D convolution blocks and a 2D transposed convolution layer for 2D up-sampling. The 2D convolution block consisted of a 3 × 3 convolution layer, batch normalization (BN), and rectified linear unit (ReLU) activation. The final output layer in FSNet was a 1 × 1 convolution layer with a Sigmoid activation function.

After automatic segmentation of the frontal sinus on CBCT images by FSNet, the CBCT scan with corresponding prediction masks cropped at the centroid of the segmentation results of the frontal sinus were used as multi-channel inputs of the SDNet designed for automatic sex determination (Fig. 2a). SDNet had 3D convolutional blocks (ConvBlocks), an anatomy-guided attention module (AGAM), 3D max-pooling (MP), and 3D global average pooling (GAP). The ConvBlock consisted of a 3 × 3 × 3 convolution layer, BN, and ReLU. The MP was used for the down-sampling of 3D feature maps. We employed 3D GAP to average each 3D feature map. Final feature vectors by a 3D GAP were fed into the output layer with the Sigmoid activation function for sex prediction. The feature maps at each level of layers were gradually increased from 16 to 32, 64, and 128 in SDNet.

For accurate sex determination from a CBCT scan, deep learning models need to capture anatomical context information related to variations in the shape and size of the frontal sinuses between males and females (Supplementary Fig. S2a–c). Attention mechanisms in deep learning are inspired by the human visual cognition system, and these are used to encourage deep learning models to focus more on the most relevant regions and ignore the background^32^. Based on this observation, we proposed AGAM in SDNet to encourage the deep learning model to focus more on the frontal sinus regions on a CBCT scan than on the background regions (Fig. 2b). AGAM was embedded in each level of the layer of SDNet to learn the anatomical context of the frontal sinus hierarchically. Frontal sinus segmentation maps ${S}_{m}\in {R}^{H\times W\times D\times 1}$ are obtained by the ROI extraction process after the inference of FDNet, where *H*, *W*, and *D* indicate the height, width, and depth of the mask maps, respectively. The 3D feature maps ${F}_{m}\in {R}^{H\times W\times D\times C}$ are acquired by a ConvBlock, where *C* indicates the number of channels in the feature maps. Then, we employ a 1 × 1 × 1 convolution layer $Con{v}_{1}$ for each ${F}_{m}$ and to obtain anatomy-guided feature maps ${A}_{m}\in {R}^{H\times W\times D\times C}$ and bottleneck feature maps ${B}_{m}\in {R}^{H\times W\times D\times C}$ as follows:

1 $${A}_{m}=Con{v}_{1}({S}_{m}), { B}_{m}=Con{v}_{1}({F}_{m})$$

To extract discriminative features ${F}_{dis}$ between ${A}_{m}$ and ${B}_{m}$, 3D attention maps (${F}_{att}\in {R}^{H\times W\times D\times C}$) are acquired as follows:

2 $${F}_{dis}=\psi \left({\sigma }_{1}\left({A}_{m}+{B}_{m}\right)\right)$$

3 $${F}_{att}= GR\left({\sigma }_{2}\left({F}_{dis}\right)\right)$$

where $\psi$ is a 1 × 1 × 1 convolution layer to extract the discriminative feature map ${F}_{dis}\in {R}^{H\times W\times D\times 1}$ and ${\sigma }_{1}$ and ${\sigma }_{2}$ are ReLU and Sigmoid activation functions, respectively. *GR* denotes a grid resampling operation to restore the dimensions of the discriminative feature map to the same as that of ${F}_{m}$ using trilinear interpolation. Finally, 3D attentive feature maps ${F}_{n}$ are acquired by elemental-wise multiplying ${F}_{m}$ and ${F}_{att}$ as follows:

4 $${F}_{n}={F}_{att}\otimes {F}_{m}$$

where $\otimes$ indicates elemental-wise multiplying. ${F}_{att}\in [ {0,1}]$, which are saliency maps, identified important regions in the feature maps and pruned the feature response to retain the activations relevant to the foreground, suppressing the background.

We used the Dice similarity coefficient (DL) and binary cross-entropy (BL) losses to train FSNet and SDNet, respectively. DL measured the overlap between the ground truth and segmentation results for the frontal sinus. DL is defined as:

5 $$DL\left(y,\widehat{y}\right)=1-2\frac{{\sum }_{i}^{n}\left({y}_{i}\times \widehat{{y}_{i}}\right)+\epsilon }{\left({\sum }_{i}^{n}{y}_{i}+{\sum }_{i}^{n}\widehat{{y}_{i}}\right)+\epsilon }$$

where $y$ and $\widehat{y}$ are ground truth and segmentation results for the frontal sinus, respectively, and $n$ is the number of pixels on CBCT images. $\epsilon$ provided numerical stability to prevent division by zero, with $\epsilon$ set to 10^–3^. BL measured the average probability error between the ground truth (actual sex) and the sex predictions. BL is defined as:

6 $$BL\left(p,\widehat{p}\right)=1- {\sum }_{i}^{N}\left({p}_{i} {log}\widehat{{p}_{i}}\right)$$

where $p$ and $\widehat{p}$ are the ground truth and probability of sex prediction, respectively. *N* is the number of CBCT scans.

FSNet was trained for 200 epochs with a mini-batch size of 16. Data augmentation was performed with rotation (− 10° to 10°), Gaussian blur (− 10% to 10%), and brightness (− 10° to 10°). Adam optimizer with a learning rate of 10^–3^ was used as the initial setting, and the learning rate was reduced by half up to 10^–6^ when the validation loss saturated for 20 epochs. SDetNet was trained for 100 epochs with a mini-batch size of 1. Adam optimizer was used with ${\beta }_{0}=0.9$ and ${\beta }_{1}=0.999$, and the learning rate was initially set to 10^–4^, which was reduced by half up to 10^–7^ when the validation loss saturated for 25 epochs. Deep learning models were implemented with Python3 and Keras with a TensorFlow backend based on an Intel i9-7900X CPU 3.3 GHz, 256 RAM, and an NVIDIA RTX A6000 GPU 48 GB.

### Performance evaluation

We used precision (PR), recall (RC), Jaccard index (JI), and F1-score (F1) to evaluate the segmentation performance of deep learning models for the frontal sinus, and area under the receiver operating characteristic curve (AUC), Brier score (BR), accuracy (ACC), specificity (SPE), sensitivity (SEN), and the polygon area metric (PAM) to evaluate its performance for sex determination. PR is calculated as the number of true positives (TP) divided by the sum of the TP and false positives (FP): ${PR}=\frac{ {TP}}{ {TP}+ {FP}}$. RC is calculated as the number of TPs divided by the sum of the TPs and false negatives (FNs) as follows: ${RC}=\frac{ {TP}}{ {TP}+ {FN}}$. JI is calculated as the intersection of the predicted segmentation and ground truth divided by the union of the two: ${JI}=\frac{ {TP}}{ {TF}+ {FP}+ {FN}}$. F1 is calculated as the harmonic mean of the PR and RC: $F1=\frac{2\times {PR}\times {RC}}{ {PR}+ {RC}}$. ACC is defined as the ratio of the number of correct sex predictions to the total number of input samples as follows: ${ACC}=\frac{ {TP}+ {TN}}{ {TF}+ {TN}+ {FP}+ {FN}}$, where TN indicates true negatives. SPE is a metric that measures a model’s ability to predict negative cases correctly and is defined as ${SPE}=\frac{ {TN}}{ {TN}+ {FP}}$. SEN, similar to RC, is a metric that measures a model’s ability to correctly predict positive cases. BR is calculated as the mean squared difference between the predicted probabilities and the actual outcomes: ${BR}=\frac{1}{N}{\sum }_{i}^{N}{\left({y}_{i}-{p}_{i}\right)}^{2}$, where *N* is the number of CBCT scans and ${y}_{i}$ and ${p}_{i}$ are the ground truth and prediction probability, respectively. AUC is calculated as the area under the receiver operating characteristic (ROC) curve, which is a plot of the true positive rate versus the false positive rate. PAM is calculated using the area of the polygon including ACC, SEN, SPE, AUC, Jaccard index (JI), and F-measure (FM) points generated in a regular hexagon^33^. The PAM is defined as:

7 $${PAM}=\frac{ {PA}}{2.59807}$$

where, the PA denotes the area of the polygon. To normalize the PAM into the [0, 1], the PA is divided by 2.59807. In terms of sex determination results, SDetNet outputs a probability within the range of 0.0 to 1.0, where females and males are classified based on the 0.5 threshold as 0 and 1, respectively. Therefore, SPE reflected the ability of the deep learning algorithm to correctly predict females and SEN the ability of the algorithm to correctly predict males. An analysis of variance (one-way ANOVA) with Scheffé post hoc tests was performed using IBM SPSS Statistics (IBM SPSS Statistics for Windows 10, Version 26.0; IBM, Armonk, New York, USA), and statistical significance (*p*-value) was set to 0.05.

### Ethics declarations

This study was performed with approval from the Institutional Review Board (IRB) of Seoul National University Dental Hospital (ERI123041). The IRB of Seoul National University Dental Hospital approved the waiver for informed consent because this was a retrospective study. The study was performed in accordance with the Declaration of Helsinki.

## Results

We compared the performances of the VGG16, ResNet101, Inception V3, EfficientNet-B5, and DenseNet201 backbones in FSNet for frontal sinus segmentation using JI, F1, PR, and RC. After frontal sinus segmentation, the sex determination performance of SDNet was compared with that of 3D ResNet, 3D DenseNet, 3D MobileNet, and 3D EfficientNet-B0 using ACU, BR, ACC, SPE, SEN, and PAM. To ensure a fair comparison, all deep learning models were run in the same computational environment.

**Figure 3:**
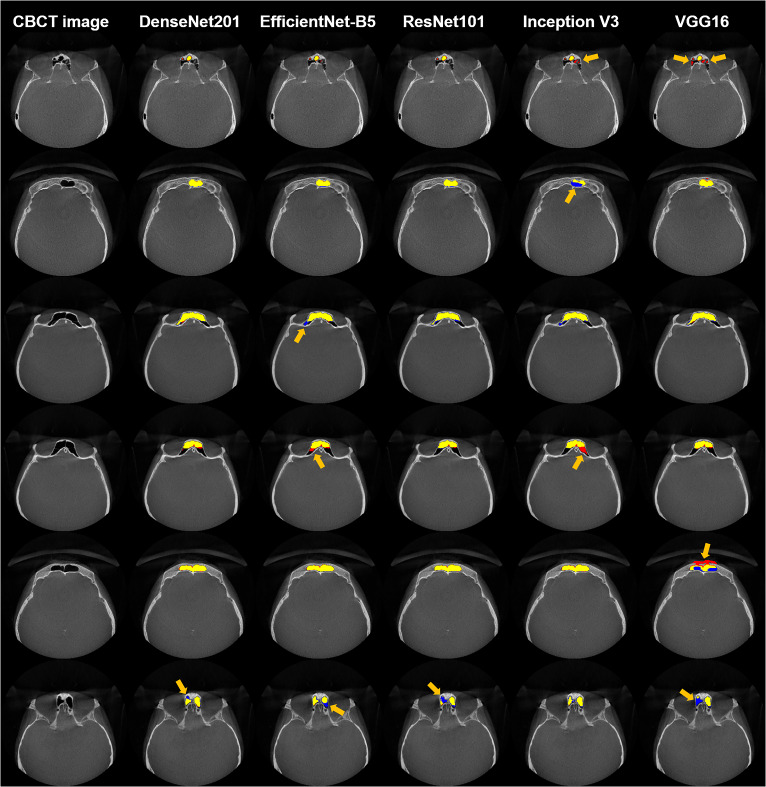
2D segmentation results from the different backbones of DenseNet201, EfficientNet-B5, ResNet101, Inception V3, and VGG16 in FSNet. Yellow, blue, and red areas present true positives, false positives, and false negatives for frontal sinus segmentation, respectively. The orange arrow indicates segmentation errors.

**Figure 4:**
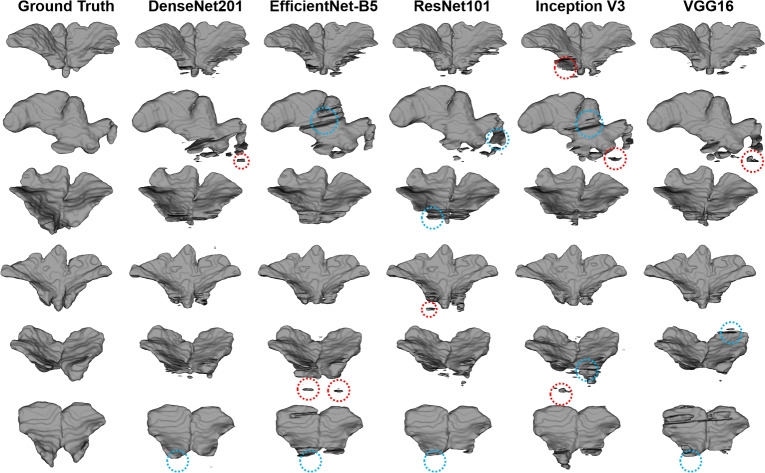
3D reconstruction of the segmentation results of the frontal sinus from different backbones including DenseNet201, EfficientNet-B5, ResNet101, Inception V3, and VGG16 in FSNet. DenseNet201 shows fewer false negatives (blue dot circles) and false positives (red dot circles) than the other backbones.

**Figure 5:**
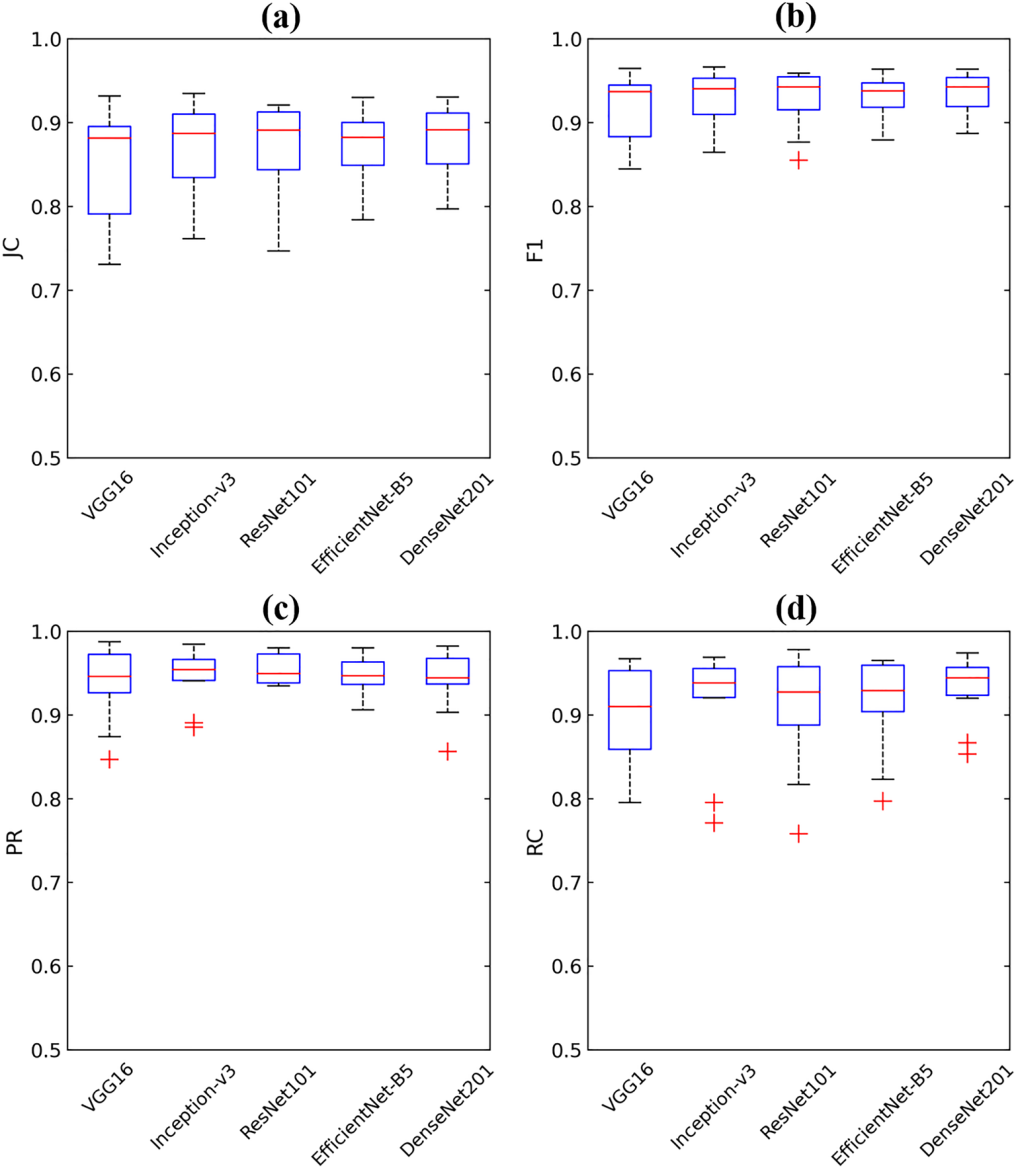
Boxplots of the segmentation performance of the frontal sinus from different backbones in FSNet. Each boxplot contains the first and third quartiles of data. Medians are located inside the boxes and are represented as red lines. Whiskers that extend above and below each box are ± 1.5 times the interquartile range (IQR), and outliers are indicated as red crosses (IQR values 1.5 or greater away from the box).

As shown in Table 2, all backbones achieved high segmentation performance of F1 values of over 0.900. for frontal sinuses on CBCT images. DenseNet201 achieved the highest JI, F1, and RC values of 0.878 ± 0.042, 0.935 ± 0.024, and 0.930 ± 0.038, respectively, indicating its superior segmentation performance for frontal sinuses. Representative segmentation results of the frontal sinus from different backbones are shown in Fig. 3. DenseNet201 exhibited the most accurate frontal sinus segmentation with more true positives (yellow), fewer false positives (red), and fewer false negatives (blue) than the other backbones. In the 3D reconstruction of frontal sinus segmentation from different backbones, DenseNet201 exhibited fewer false negatives (blue circles) and false positives (red circles) than the other backbones, as shown in Fig. 4. Boxplots of the segmentation performance of the frontal sinuses from different backbones are shown in Fig. 5. ROC and BR curves for the sex determination performance of SDetNet according to segmentation results generated by different backbones in FSNet are shown in Fig. 9a and d, respectively.

**Table 2:** Comparison of the segmentation performance of different backbones in FSNet.

Backbones	JI	F1	PR	RC
VGG16	0.850 ± 0.064	0.917 ± 0.038	0.937 ± 0.043	0.902 ± 0.059
Inception V3	0.865 ± 0.059	0.927 ± 0.035	0.945 ± 0.031	0.912 ± 0.067
ResNet101	0.868 ± 0.058	0.928 ± 0.035	0.955 ± 0.018	0.906 ± 0.067
EfficientNet-B5	0.868 ± 0.048	0.929 ± 0.028	0.946 ± 0.022	0.914 ± 0.056
DenseNet201	0.878 ± 0.042	0.935 ± 0.024	0.941 ± 0.035	0.930 ± 0.038

Segmentation performance is presented as mean ± standard deviation.

**Figure 6:**
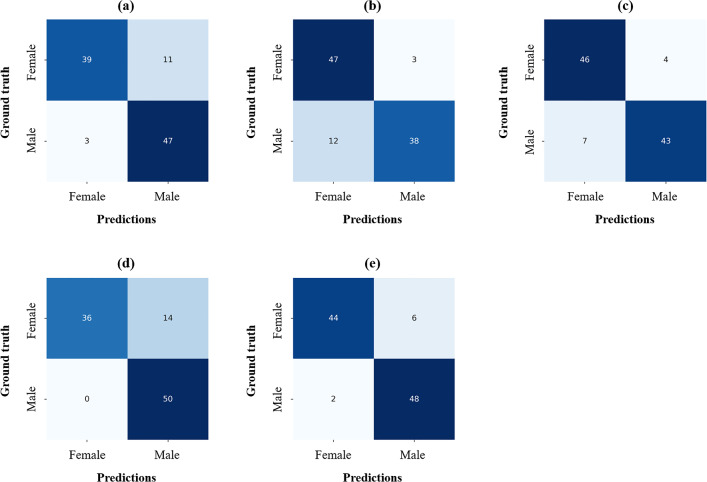
Confusion matrices for sex determination performance of SDetNet according to segmentation results generated by the different backbones in FSNet. (**a**–**e**) Results for VGG16, Inception V3, ResNet101, EfficientNet-B5, and DenseNet201, respectively.

From the quantitative results of the sex determination according to segmentation results from different backbones in FSNet, SDetNet with ROIs extracted by DenseNet201 achieved superior AUC, ACC, BR, SPE, SEN, and PAM values of 0.979, 0.920, 0.063, 0.960, 0.880, and 0.828, respectively, for sex determination (Table 3). Polygon area graphs are shown in Supplementary Fig. S3. Although SDetNet using ROIs extracted by the other backbones in FSNet showed comparable performance in sex determination, it achieved slightly lower AUC and ACC values than SDetNet using DenseNet201 (Table 3). Confusion matrices for the sex determination performance of SDetNet according to segmentation results generated by different backbones in FSNet are shown in Fig. 6.

**Table 3:** Sex determination performance of SDetNet according to segmentation results generated by different backbones in FSNet.

Backbones	AUC	ACC	BR	SPE	SEN	PAM
VGG16	0.946	0.860	0.098	0.780	0.940	0.716
Inception V3	0.968	0.850	0.093	0.940	0.760	0.680
ResNet101	0.981	0.890	0.063	0.920	0.860	0.718
EfficientNet-B5*	0.969	0.860	0.105	0.720	1.000	0.763
DenseNet201	0.979	0.920	0.063	0.960	0.880	0.828

*Significant difference for predicted probability between ResNet101 and EfficientNet-B5 (p-value < 0.05).

**Figure 7:**
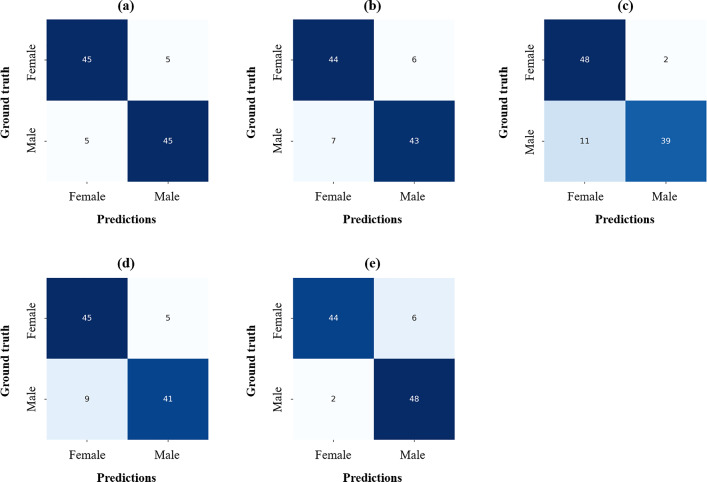
Confusion matrices for sex determination performance of different 3D CNNs according to segmentation results generated by DenseNet201. (**a**–**e**) Results of Simple 3D CNN, 3D InceptionNet, 3D DenseNet, 3D ResNet, and SDetNet, respectively.

The sex determination performance of SDetNet was compared quantitatively with those of the other 3D CNNs (Table 4), with the ROI of the frontal sinus extracted using DenseNet201. SDetNet outperformed the other 3D CNNs by obtaining the highest AUC, ACC, BR, SPE, and PAM values of 0.979, 0.920, 0.063, 0.960, and 0.828, respectively. Compared with the second-highest performing backbone, the AUC, ACC, BR, and PAM values of SDetNet were enhanced by 0.002, 0.020, 0.032, and 0.065 better, respectively. Polygon area graphs are shown in Supplementary Fig. S4. The confusion matrix for the sex determination performance of the different 3D CNNs is shown in Fig. 7. The ROC and BR curves for the sex determination performance of the different 3D CNNs according to segmentation results generated by DenseNet201 are shown in Fig. 9b and e, respectively.

**Table 4:** Sex determination performance of different 3D CNNs according to segmentation results generated by DenseNet201.

Backbones*	AUC	ACC	BR	SPE	SEN	PAM
Simple 3D CNN	0.964	0.900	0.098	0.900	0.900	0.785
3D InceptionNet	0.950	0.870	0.105	0.880	0.860	0.727
3D DenseNet	0.977	0.870	0.091	0.960	0.780	0.717
3D ResNet	0.934	0.860	0.121	0.900	0.820	0.705
SDetNet	0.979	0.920	0.063	0.960	0.880	0.828

*No significant difference for predicted probability between backbones.

**Figure 8:**
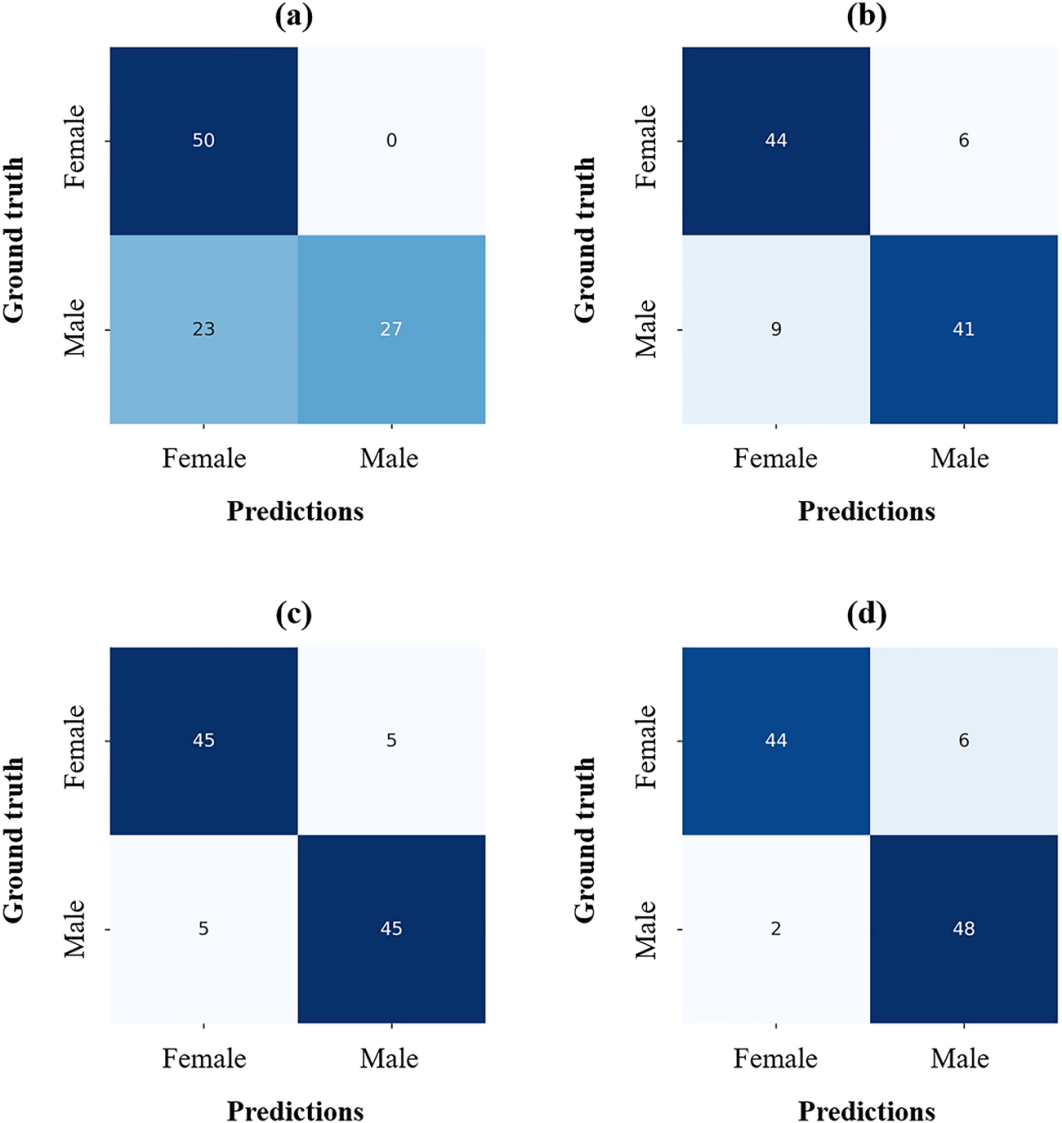
Confusion matrices for sex determination performance of each component in SDetNet. (**a**–**d**) Results of CBCT scan, Mask images, CBCT scan + Mask images, and CBCT scan + Mask images + AGAM, respectively.

**Figure 9:**
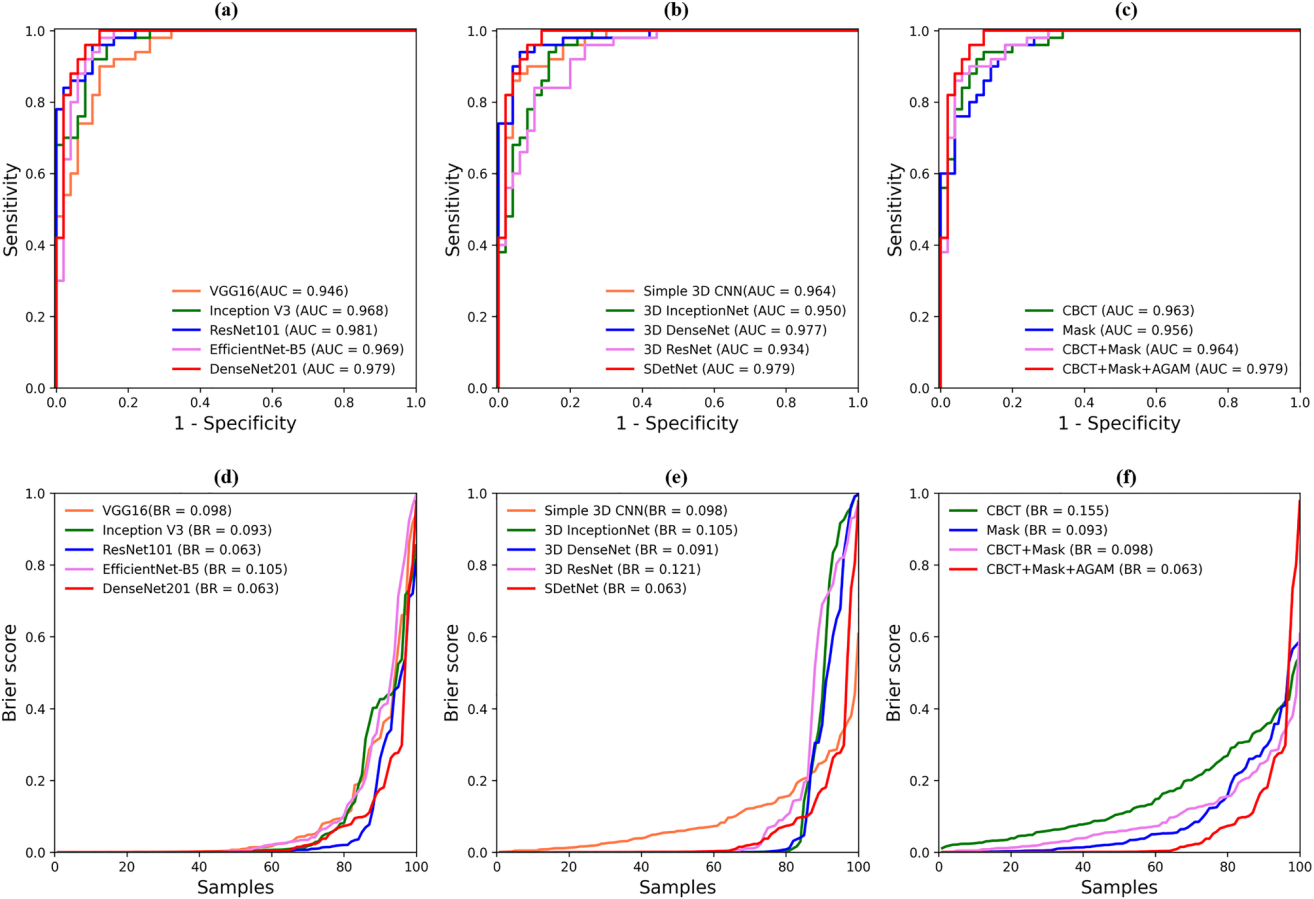
Receiver operating characteristic (ROC) and Brier score (BR) curves for sex determination performance. (**a**, **d**) are ROC and BR curves showing the sex determination performance of SDetNet according to segmentation results generated by different backbones in FSNet, respectively. (**b**, **e**) are ROC and BR curves for the sex determination performance of different 3D CNNs according to segmentation results generated by DenseNet201, respectively. (**c**, **f**) are ROC and BR curves for the sex determination performance of each component of SDetNet.

Ablation studies were performed to demonstrate the effectiveness of MSI and AGAM in SDetNet (Table 5). For sex determination, SDetNet without MSI (without mask images) and AGAM obtained lower ACC, BR, SEN, and PAM values of 0.770, 0.155, 0.540, and PAM, respectively, than SDetNet using both CBCT scans and mask images. In addition, sex determination performance was further improved by embedding AGAM in SDetNet as evidenced by AUC, ACC, BR, SPE, and PAM values of 0.964, 0.920, 0.098, 0.900, and 0.785 to 0.979, 0.920, 0.063, 0.960, and 0.828, respectively. Polygon area graphs are shown in Supplementary Fig. S5. Figure 8 shows the confusion matrices for the sex determination performance of each component in SDetNet. The ROC and BR curves for the sex determination performance of each component in SDetNet are shown in Fig. 9c and f, respectively.

**Table 5:** Ablation experimental results of each component of SDetNet on the test dataset.

CBCT scan	Mask images	AGAM	AUC	ACC	BR	SPE	SEN	PAM
✓			0.963	0.770	0.155	1.000	0.540	0.510
	✓		0.956	0.850	0.093	0.880	0.820	0.688
✓*	✓		0.964	0.900	0.098	0.900	0.900	0.785
✓^†^	✓	✓	0.979	0.920	0.063	0.960	0.880	0.828

*Significant difference for predicted probability between only used CBCT scan and CBCT scan + Mask images (p-value < 0.05); ^†^Significant difference for predicted probability between only used CBCT scan and CBCT scan + Mask images + AGAM (p-value < 0.05).

## Discussion

Sex determination based on skeletal remains is essential in forensic investigations and human identification after mass disasters, homicides, and accidents^1,2^. Manual morphological measurement and analysis of the skeletal remains are widely used to determine the sex of individuals^4–9,26^. In particular, the unique patterns of the frontal sinuses in males and females on CBCT scans make these sinuses an important and reliable tool for sex determination^16,20,34^. Recently, deep learning-based methods have been applied for forensic investigation to predict sex from CT or CBCT scans^20,24,25,34^. However, previous methods required several steps including manual segmentation of the skull, manual measurement of dental parameters, feature extraction, and classification, which are all labor-intensive, time-consuming, and error-prone steps. In this study, we proposed SDetNet to automatically and accurately determine sex from a CBCT scan by capturing subtle differences in shapes and sizes of the frontal sinus on CBCT scans between males and females.

The segmentation performance of backbones such as VGG16, ResNet101, Inception V3, EfficientNet-B5, and DenseNet201 in FSNet was compared. DenseNet201 outperformed the other backbones for frontal sinus segmentation on CBCT images (Table 2), with much higher RC values than the other backbones. As shown in Figs. 3 and 4, segmentation models exhibited false positives of segmentation of the frontal sinus with invading ethmoid cells. The ethmoid sinuses are the only paranasal sinuses not formed by a single cavity, making them more complex. The anterior cranial fossa and the frontal bone limit the ethmoid cells superiorly, and thus, ambiguous structures between the ethmoid cells and the frontal sinus yield false positives. Additionally, inflammation of the frontal sinus can manifest as a thickening of the mucous membrane. Mucosal thickening may obscure clear borders, making automatic segmentation of the frontal sinus more challenging on CBCT images. Compared with backbones in FSNet, SDetNet using segmentation masks generated by DenseNet201 achieved superior sex determination performance (Table 3). The sex determination performance of SDetNet was affected by the segmentation quality of the frontal sinus on CBCT images.

The rationale for choosing the five backbones can be summarized as follows: (1) The selected backbones have been extensively validated on benchmark datasets and have shown remarkable performance in various tasks, including medical image classification, object detection, and segmentation^35,36^. Their proven performance provides a strong foundation for FSNet, potentially enhancing its segmentation performance and reliability. (2) Each backbone has different architectural designs and principles that can affect the prediction performance. VGG16 increases the depth of an architecture using 3 × 3 convolution layers. ResNet101 is designed based on residual learning that facilitates deep learning without degradation. DenseNet201 adopts the reuse of feature maps to enhance information flow. Inception V3 uses multi-scale convolution layers that can capture information at various resolutions. EfficientNet leverages a compound scaling method, balancing the depth, width, and resolution of convolution layers, showcasing parameter usage and computational performance efficiency.

Compared with different 3D CNNs, SDetNet achieved the highest sex determination performance (Table 4). SDetNet with DenseNet201 achieved a superior performance owing to three key factors. First, ROIs including frontal sinus regions on CBCT images were automatically extracted by FSNet and used as the input volume in SDetNet. Using ROIs as input volume can help a deep learning model focus on the frontal sinus regions on a CBCT scan and determine sex well, without having to consider larger anatomical structures. Second, DenseNet201 in FSNet alleviated the vanishing gradient problem by connecting each convolutional layer to every other layer. This allowed the deep learning model to learn more complex features and improved its segmentation performance. Finally, AGAM embedded in SDetNet was designed to focus on anatomical features of the frontal sinus for sex determination from a CBCT scan. The proposed AGAM and MSI improved the sex determination performance, and their effectiveness was demonstrated by an ablation study (Table 5). The primary reason for this improvement was that the shape and size of the frontal sinuses differ slightly between males and females, and SDetNet could learn the anatomical context information about subtle differences in shape and size of the frontal sinuses between males and females using AGAM and MSI.

Previous studies have used 2D slices from multiplanar reconstructions to measure the volume of the frontal sinus cavity^11^. However, this approach can be challenging due to the high variability in size, shape, and asymmetry of the cavity. Other studies segmented and reconstructed the frontal sinuses in 3D, and performed calculations on the reconstructed volume after exporting different views (frontal, lateral, basal)^19,20^. This approach avoids the loss of information and allows for more accurate measurements. Nevertheless, the accuracy of previous studies at predicting sex correctly based on analysis of images of the frontal sinuses ranged between 60 and 80%^13,19,20,34,37^. Our proposed SDetNet is a fully automatic and accurate sex determination method that consists of FSNet and SDNet, achieving an AUC, ACC, BR, and PAM of 0.979, 0.920, 0.063, and 0.828. SDetNet does not require any additional processes such as manual segmentation or analysis, dental parameters, or feature selection.

The following issues will be addressed in future studies to improve the sex determination performance of SDetNet. First, our dataset was built using CBCT scans from a patient group with a non-uniform age distribution. To improve the accuracy of our SDetNet for sex determination at all ages, we need to collect additional datasets with a uniform age distribution. Second, it would be valuable to assess the model’s performance on a more diverse and larger dataset to validate its generalizability. This study relied on a CBCT dataset from a single organization in South Korea, which may not be generalizable to other populations or organizations. Therefore, further research is needed to train and evaluate SDetNet using CBCT datasets acquired from individuals of diverse ethnicities using various devices at multiple organizations. Finally, we applied several exclusion criteria when selecting CBCT scans. In future studies, we plan to improve the generalizability and clinical efficacy of SDetNet using large-scale panoramic radiographs from individuals of all ages with fewer exclusion criteria.

## Conclusions

In this study, we proposed SDetNet for automatic and accurate sex determination from a CBCT scan. SDetNet was designed as a two-stage network to learn the anatomical context information of the frontal sinuses between males and females by embedding MSI and AGAM in an end-to-end manner. The experimental results showed the SDetNet outperformed existing 3D CNNs for sex determination from CBCT scans. Furthermore, we demonstrated the effectiveness of MSI and AGAM of SDetNet by an ablation study, which substantially improved sex determination from CBCT scans. SDetNet is a fully automatic and accurate sex determination method, that will likely improve the workflow of forensic investigations and individual identification in clinical settings. In future studies, we plan to improve the generalizability and clinical efficacy of SDetNet by using CBCT scans of the frontal sinuses of individuals of varied ethnicities from diverse populations collected by multiple organizations using various devices.

### Supplementary Information

Supplementary Figures.

## Data Availability

The datasets generated and/or analyzed during the current study are not publicly available due to restrictions of the Institutional Review Board (IRB) of Seoul National University Dental Hospital to protect patients’ privacy but are available from the corresponding author on reasonable request.
