# Supplementary material for: Automatic segmentation and classification of frontal sinuses for sex determination from CBCT scans using a two-stage anatomy-guided attention network
Source: Sci Rep. 2024 May 23;14:11750. doi: 10.1038/s41598-024-62211-y (PMC11116511; doi:10.1038/s41598-024-62211-y)
Supplement: Supplementary file 1 — Supplementary Figures. [file 41598_2024_62211_MOESM1_ESM.pdf]

Supplementary Materials

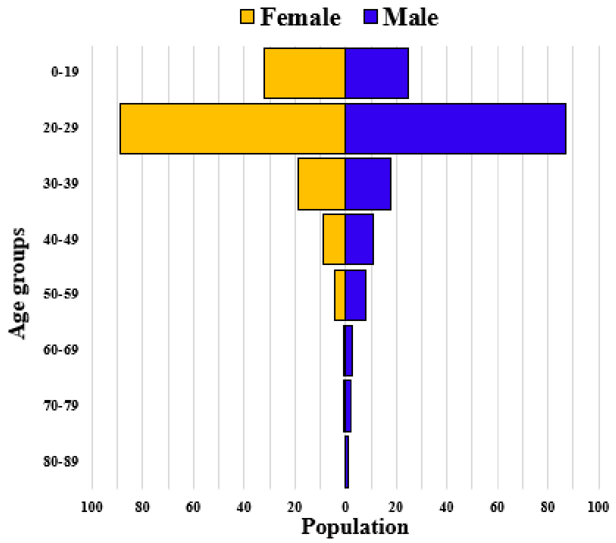

Figure S1. Population pyramid of our dataset.

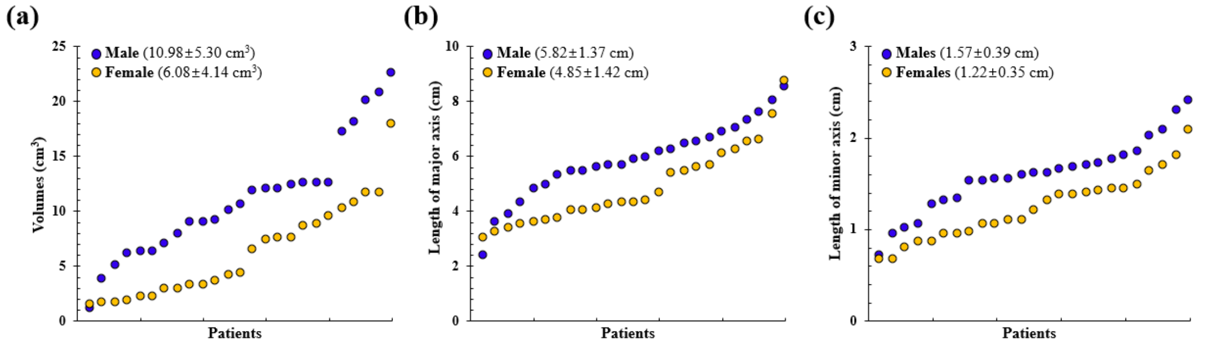

Figure S2. (a)-(c) Dot plots of volume, length of the major axis, and length of the minor axis of the frontal sinuses, respectively, in males and females.

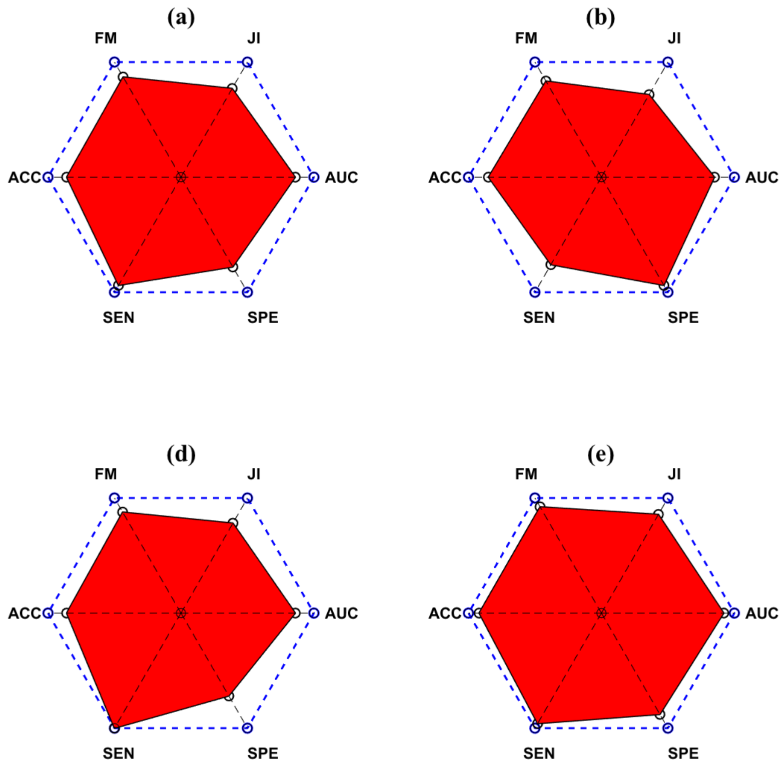

Figure S3. Polygon area graphs of SDetNet according to segmentation results generated by different backbones in FSNet. (a)-(e) Results for VGG16, Inception V3, ResNet101, EfficientNet-B5, and DenseNet201, respectively. ACC, FM, JI, AUC, SPE, and SEN denote accuracy, F-measure, Jaccard index, the area under the receiver operating characteristic curve, specificity, and sensitivity, respectively.

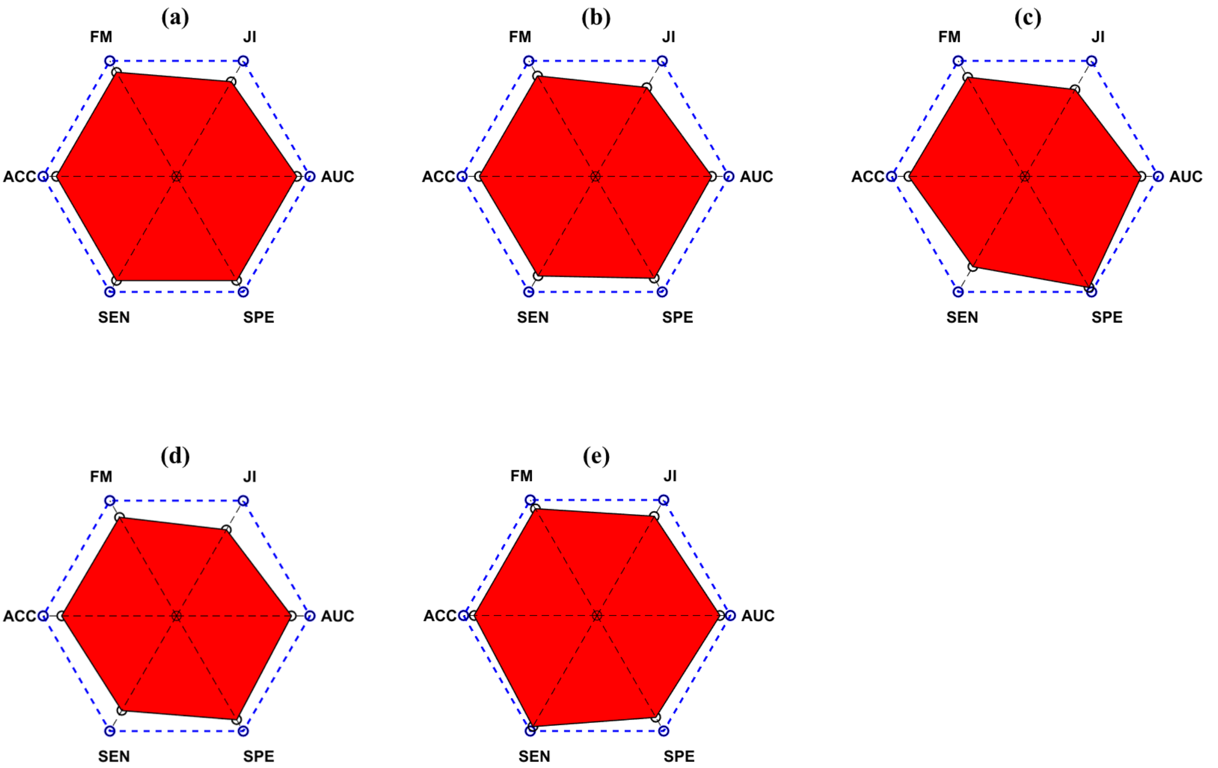

Figure S4. Polygon area graphs of different 3D CNNs according to segmentation results generated by DenseNet201. (a)-(e) Results for VGG16, Inception V3, ResNet101, EfficientNet-B5, and DenseNet201, respectively. ACC, FM, JI, AUC, SPE, and SEN denote accuracy, F-measure, Jaccard index, the area under the receiver operating characteristic curve, specificity, and sensitivity, respectively.

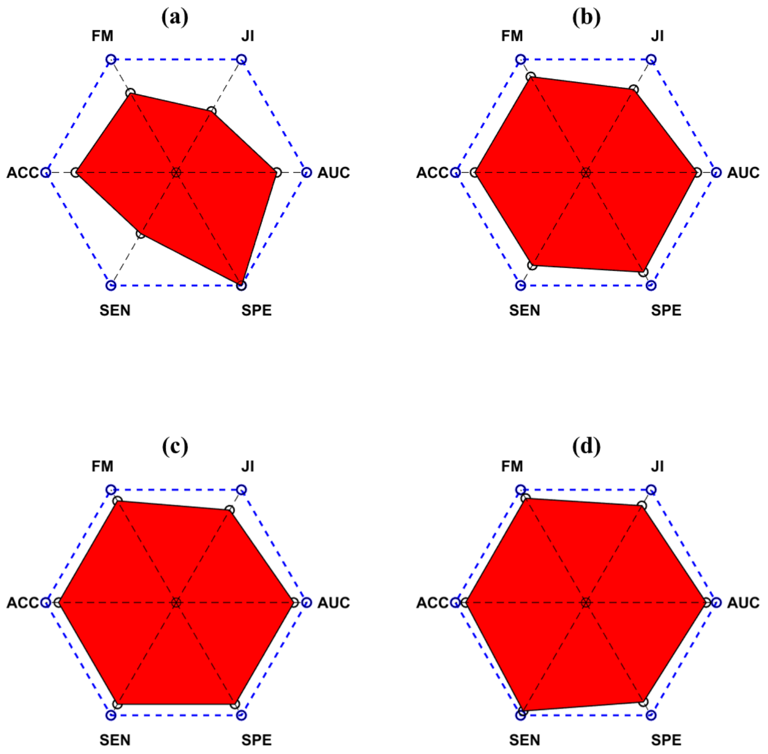

Figure S5. Polygon area graphs of each component of SDetNet. (a)-(d) Results of CBCT scan, Mask images, CBCT scan + Mask images, and CBCT scan + Mask images + AGAM, respectively. ACC, FM, JI, AUC, SPE, and SEN denote accuracy, F-measure, Jaccard index, the area under the receiver operating characteristic curve, specificity, and sensitivity, respectively.
